# Cyst in the Mist: A Surgical Perspective on Mesenteric Cysts

**DOI:** 10.7759/cureus.88074

**Published:** 2025-07-16

**Authors:** Amrutha T, Balaka Kuladeep, Akshatha Sujay Mendon, Parikshith H K

**Affiliations:** 1 General Surgery, Vydehi Institute of Medical Sciences and Research Centre, Bengaluru, IND

**Keywords:** cystic lesion, exploratory laparotomy, histopathology, lower abdominal mass, mesenteric cyst

## Abstract

A 32-year-old woman presented with dull, non-radiating lower abdominal pain that had persisted for two months and intensified over the preceding 15 days. She reported no gastrointestinal or urinary symptoms. Abdominal examination revealed a soft, non-tender, freely mobile mass measuring approximately 10 × 15 cm in the right iliac fossa. Differential diagnoses included ovarian cyst, uterine fibroid, and mesenteric cyst. Routine blood investigations were within normal limits. Ultrasonography revealed a large anechoic cystic lesion in the right iliac fossa and a smaller pelvic cyst. A contrast-enhanced CT scan confirmed the presence of a well-defined, unilocular, thin-walled mesenteric cyst measuring 12 × 15 cm, along with a smaller ovarian cyst measuring 4.5 × 3 cm. The patient underwent elective exploratory laparotomy, during which both cysts were successfully excised. Histopathological examination confirmed benign cysts lined by flat to cuboidal epithelium. Postoperative recovery was uneventful. This case highlights the importance of early imaging and timely surgical intervention in achieving accurate diagnosis and preventing complications associated with mesenteric cysts.

## Introduction

Mesenteric cysts are rare intra-abdominal lesions, with a reported incidence of approximately one in 250,000 adult hospital admissions [1]. They can occur anywhere along the mesentery but are most commonly found in the small bowel mesentery, particularly the ileum. These cysts are often asymptomatic or present with nonspecific symptoms such as abdominal pain, distension, or a palpable mass [2,3].

The pathogenesis of mesenteric cysts remains unclear. Proposed mechanisms include congenital malformations of the lymphatic system, trauma, neoplasia, or infections leading to lymphatic obstruction [4]. Although many cases go undiagnosed until imaging or surgery is performed, ultrasonography and contrast-enhanced CT (CECT) are highly effective tools for identifying and characterizing these cysts [5].

Complete surgical excision remains the gold standard for treatment, as it prevents recurrence and complications such as hemorrhage, torsion, infection, or bowel obstruction. Both open and minimally invasive approaches - including laparoscopic and robotic techniques - have shown favorable outcomes, with the latter offering shorter hospital stays and faster recovery times [6,7].

We report a rare case of a large mesenteric cyst in an adult female, coexisting with an ovarian cyst, which was diagnosed through imaging and successfully managed with surgical excision via laparotomy. This case underscores the importance of including mesenteric cysts in the differential diagnosis of abdominal masses in women of reproductive age.

## Case presentation

A 32-year-old woman presented to the surgical outpatient department with complaints of dull, aching lower abdominal pain for the past two months. The pain was non-radiating and had worsened over the preceding 15 days. She denied nausea, vomiting, fever, urinary disturbances, loss of appetite, or weight loss. Her past surgical history was significant for a lower-segment cesarean section performed 11 years prior, and she also reported irregular menstrual cycles.

On examination, she was conscious, oriented, and hemodynamically stable. General physical examination was unremarkable. Abdominal examination revealed a soft, non-tender, freely mobile mass measuring approximately 10 × 15 cm in the right iliac fossa. Notably, the size of the mass decreased on leg-raising, suggesting an intraperitoneal origin, with a positive Tillaux sign. Other systemic examinations were within normal limits.

**Figure 1:**
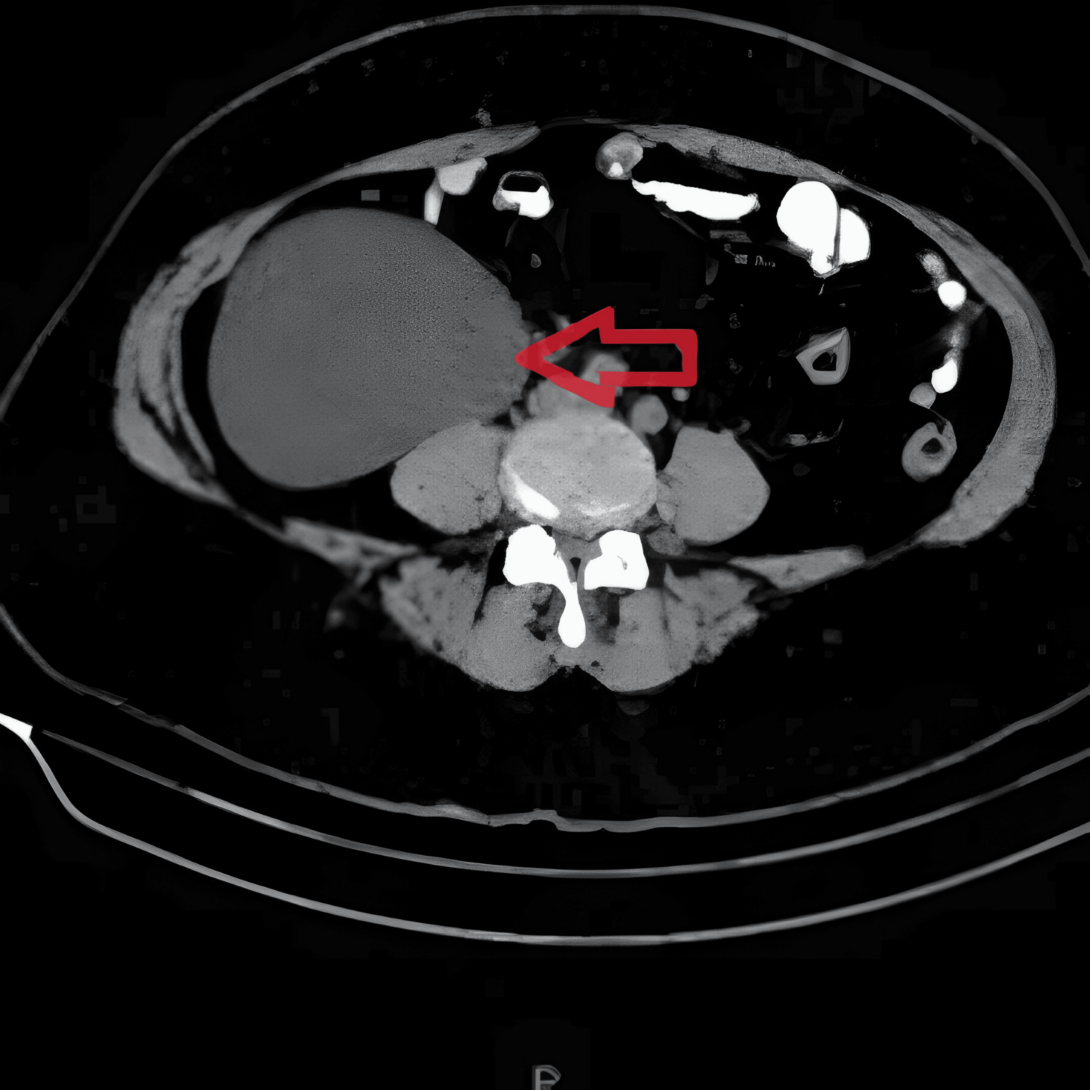
Axial view of CECT showing a right mesenteric cyst (arrow) CECT, contrast-enhanced CT

**Figure 2:**
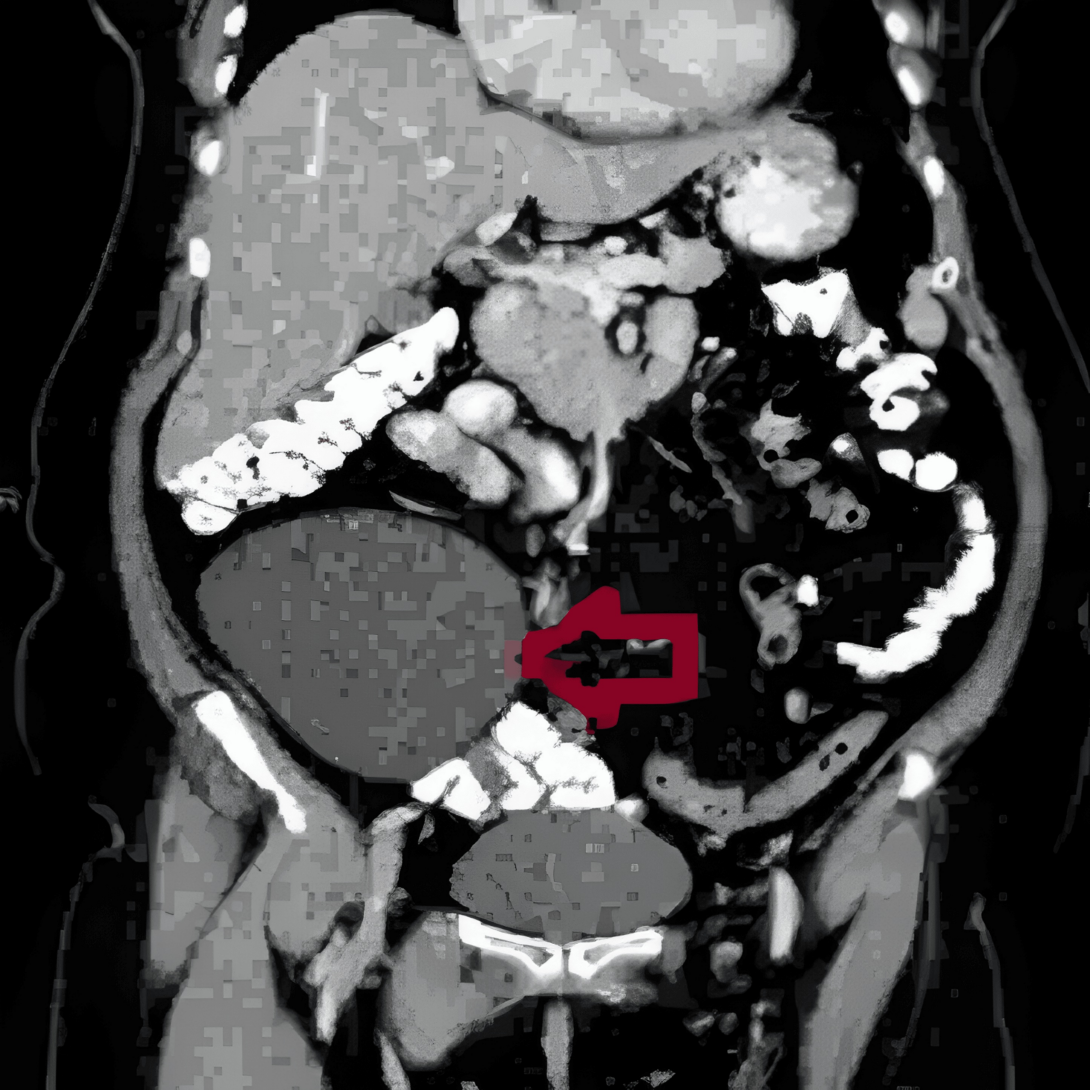
Coronal view of CECT demonstrating the mesenteric cyst (arrow) CECT, contrast-enhanced CT

Routine blood investigations were unremarkable. Ultrasonography of the abdomen and pelvis revealed a large, well-defined, anechoic cystic lesion measuring 12 × 15 cm in the right iliac fossa, suggestive of a mesenteric cyst, along with a smaller 4 × 3 cm cystic lesion arising from the right ovary, suggestive of an ovarian cyst. A CECT of the abdomen and pelvis confirmed a unilocular, thin-walled, non-enhancing cyst located within the mesentery of the right lower abdomen, as well as a 4.5 × 3 cm cyst originating from the right ovary (Figure 1, Figure 2).

**Figure 3:**
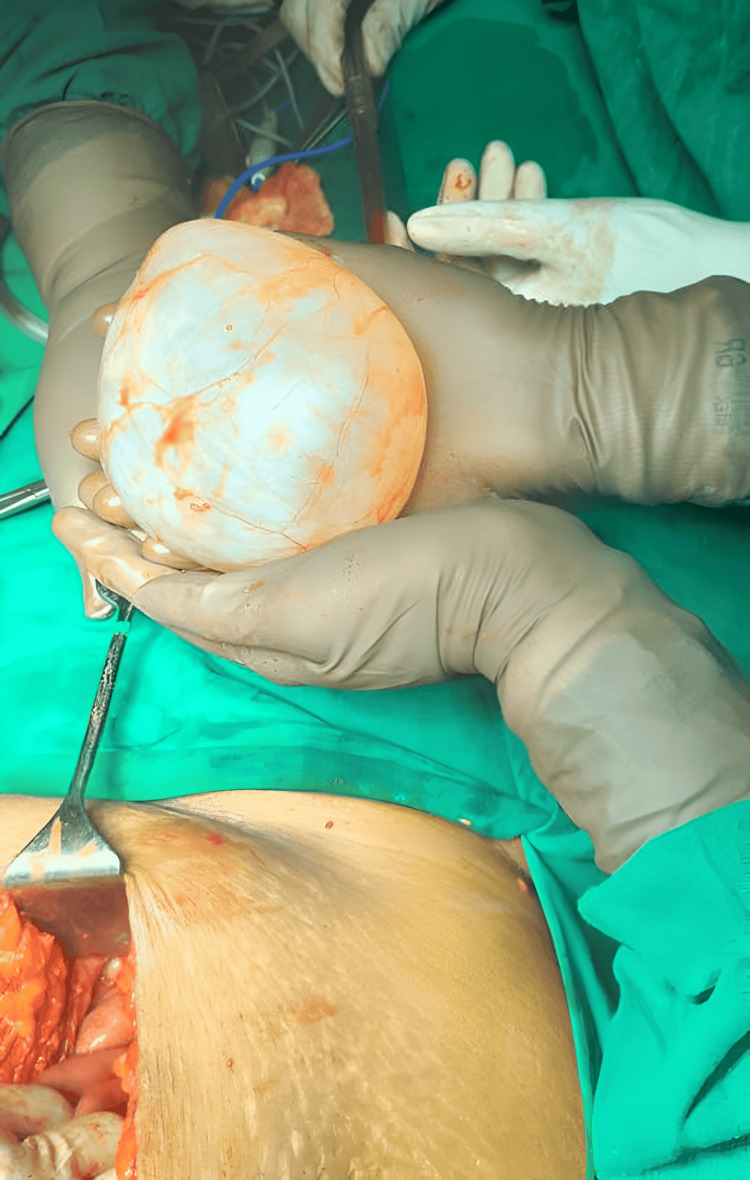
Intraoperative image of the excised mesenteric cyst

**Figure 4:**
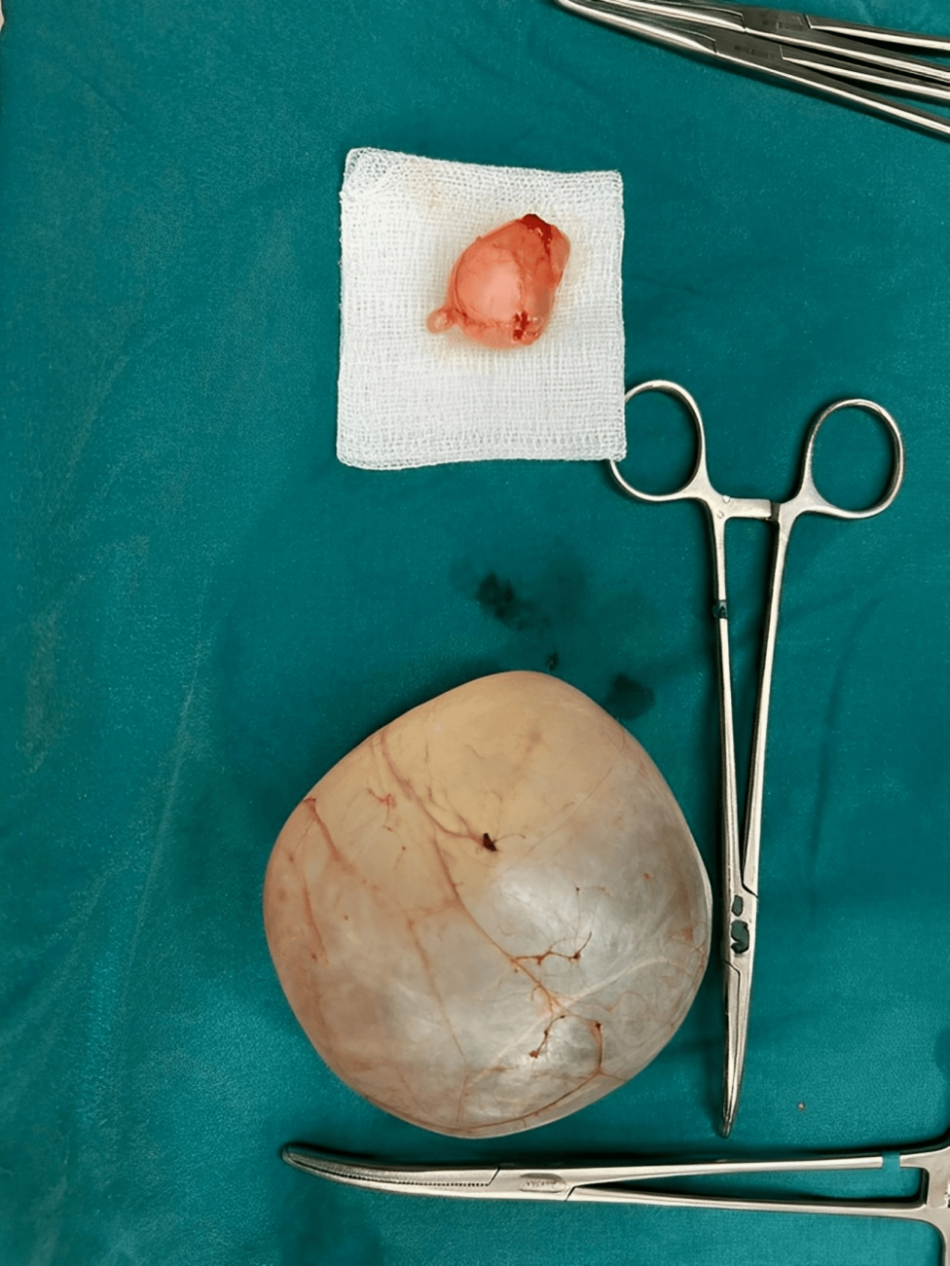
Excised mesenteric and ovarian cysts

The patient underwent an elective exploratory laparotomy. Intraoperatively, a large cystic lesion was identified arising from the mesentery of the ileum, without invasion into adjacent bowel loops or vascular structures. An additional cyst was noted on the right ovary. Complete surgical excision of both cysts was performed successfully (Figure 3, Figure 4). The patient tolerated the procedure well, with no intraoperative or postoperative complications.

Gross pathological examination revealed two cystic specimens. Microscopic evaluation showed that both cysts were lined by a single layer of bland, flat to cuboidal epithelial cells, with no evidence of atypia or malignancy, confirming the diagnoses of a benign mesenteric cyst and a benign serous ovarian cyst.

Her postoperative course was uneventful. Oral intake was gradually resumed, and she was discharged with instructions for regular follow-up. During follow-up, she remained asymptomatic, with no clinical signs of recurrence.

## Discussion

Mesenteric cysts are uncommon intra-abdominal lesions that can arise anywhere along the mesentery, most frequently involving the small intestine. Although their exact etiology remains unclear, proposed mechanisms include developmental anomalies of lymphatic vessels, trauma, neoplasia, or degenerative changes [2]. Despite their benign nature, these cysts pose a diagnostic challenge due to their rarity and nonspecific clinical manifestations.

In adults, mesenteric cysts typically present as asymptomatic abdominal masses or with vague symptoms such as pain, discomfort, or distension, as seen in our patient. The mobility and compressibility of the mass on physical examination may provide clues to its intraperitoneal origin. However, due to the overlap in presentation with gynecologic and gastrointestinal conditions, imaging plays a critical role in evaluation [6].

Ultrasound is a valuable initial modality for characterizing cystic abdominal lesions, while CECT offers superior delineation of anatomical relationships and helps exclude malignancy or vascular involvement [5]. In this case, both ultrasonography and CECT were instrumental in confirming the diagnosis and differentiating the mesenteric cyst from the concurrent ovarian cyst.

Definitive treatment involves complete surgical excision. While laparoscopic techniques are increasingly preferred due to their minimally invasive nature and favorable recovery profiles, open laparotomy remains appropriate for large cysts or when anatomical complexity is anticipated [6,7]. Complete removal is essential, as incomplete excision increases the risk of recurrence and complications such as torsion, hemorrhage, or infection [4].

Histopathological examination typically reveals cysts lined by a single layer of flat to cuboidal epithelium without dysplasia or malignant transformation, confirming their benign nature, as demonstrated in this case.

Given the potential for diagnostic uncertainty and clinical overlap, particularly in reproductive-age females, mesenteric cysts should be included in the differential diagnosis of lower abdominal masses. Early identification and complete excision are key to ensuring an excellent prognosis and minimizing morbidity.

## Conclusions

Mesenteric cysts are infrequent but clinically significant intra-abdominal lesions that should be considered in the differential diagnosis of lower abdominal masses, especially in women of reproductive age. Their presentation is often nonspecific, necessitating a high index of suspicion and appropriate imaging for accurate diagnosis. Complete surgical excision remains the cornerstone of management, essential for preventing recurrence and complications such as rupture, infection, or intestinal obstruction. This case highlights the importance of timely recognition and intervention, which contributes to favorable outcomes in the majority of patients.
